# Multi-omics analysis of the potential association between metabolic features of hypothyroidism in the first half of pregnancy and Th17/Treg balance

**DOI:** 10.3389/fimmu.2025.1656946

**Published:** 2026-01-07

**Authors:** Yixin Wang, Yajuan Xu, Jingjing Li, Miao Zhang, Zongzong Sun, Chenchen Zhang, Pengkun Lin

**Affiliations:** Department of Obstetrics and Gynaecology, The Third Affiliated Hospital of Zhengzhou University, Zhengzhou, Henan, China

**Keywords:** BID, first half of pregnancy, hypothyroidism, metabolomics, proteomics, sphingosine, Th17/Treg balance

## Abstract

The aim of this study is to conduct a multi-omics investigation of the metabolic profile of hypothyroidism in the first half of pregnancy and its correlation with the Th17/Treg balance.30 pregnant women with hypothyroidism (hypothyroidism group) and 30 healthy pregnant women (control group) in the first half of pregnancy were included. Results showed that sphingosine (Sph) and BH3 interacting domain death agonist (BID) were significantly upregulated in hypothyroidism group. Sphingolipid signaling pathway was the significantly enriched pathway. Sph and BID in cord blood of hypothyroidism group were also higher. An increase was shown in the Th17/Treg ratio and Th17 cells, and a decrease shown in Treg cells in the hypothyroidism group. The incidence of gestational diabetes mellitus in the hypothyroidism group was significantly higher. Sph and BID were positively correlated with Th17/Treg ratio. Sph levels were positively correlated with Th17 percentage and gestational diabetes.In conclusion, using a multi-omics approach, we identified distinct metabolic alterations in women with hypothyroidism in the first half of pregnancy, characterized by elevated levels of sphingosine and BID and a disrupted Th17/Treg balance. These changes may contribute to the pathogenesis of early gestational hypothyroidism, offering new theoretical insights into its underlying mechanisms.

## 1 Introduction

Hypothyroidism during pregnancy is a common endocrine disorder with a prevalence of 3–5% (1). The fetal thyroid gland is formed at 12 weeks of gestation; however, it is only at 18–20 weeks of gestation that the fetal thyroid gland produces the amount of thyroxine required to meet the needs of fetal growth and development (2). Therefore, during the first half of gestation (i.e., ≤20 weeks), thyroxine supplied by the mother to the fetus is necessary for normal fetal development (3). Patients with hypothyroidism in pregnancy are more likely to have other pregnancy complications, such as gestational diabetes mellitus and gestational hypertension, compared to pregnant women without hypothyroidism (4, 5). Also, women who develop hypothyroidism during pregnancy have an increased risk of adverse pregnancy outcomes, such as miscarriage, placental abruption, preeclampsia, and preterm labor (6).

Several studies have shown that thyroid hormone levels affect metabolic homeostasis (7, 8). It has been reported that hypothyroidism is associated with alterations in metabolic pathways such as sphingolipid metabolism, glycerophospholipid metabolism, and fatty acid β-oxidation in the liver (9–11). In the thyroid gland, sphingolipid metabolites—particularly sphingosine and sphingosine-1-phosphate (S1P)—modulate calcium signaling, which in turn influences thyroid cell proliferation, TSH receptor function, TSH-mediated responses, iodine efflux, and other key thyroid activities. Previous studies, including reports from our research group, have also shown that thyroid hormones can regulate the sphingolipid synthesis pathway (12). Our earlier work on hypothyroidism during pregnancy demonstrated altered plasma metabolic profiles in affected women, with those in late pregnancy showing disrupted sphingolipid metabolism and elevated sphingomyelin (SM) (d36:1) as a characteristic metabolic signature (8, 13). Although metabolic changes in hypothyroidism have attracted growing research interest in recent years, the relationship between thyroid dysfunction and altered metabolism remains poorly understood, and the specific mechanisms underlying their interaction are still unclear. In the body, metabolites can act as substrates, coenzymes, inhibitors, or activators of intra- and extracellular proteins to participate in a variety of cellular processes and play a role in various pathological processes (14). In a study by Xia et al., plasma proteomics and metabolomics revealed that a combination of proteins (APOL1 and ITIH4) and metabolites (cortisol and cortisone) could better discriminate between hypothyroid and healthy individuals (6). However, there is a paucity of multi-omics studies on hypothyroidism that occurs in the first half of pregnancy.

When iodine intake is adequate, autoimmune thyroid disease is the most common cause of hypothyroidism during pregnancy (15). The balance between helper T-cells 17 (Th17) and regulatory T cells (Tregs) is important for maintaining the immune microenvironment during normal pregnancy (16). Th17 cells are derived from the initial T cells in response to high levels of interleukin-6 (IL-6) and TNF-β, which elicit autoimmune and inflammatory responses in the body, while Treg cells have strong anti-inflammatory and immunomodulatory capabilities (17, 18). Studies have shown that the Th17/Treg balance is regulated by cytokines and that an abnormal balance is involved in autoimmune diseases, gestational diabetes (19), and preeclampsia (20). Th17/Treg homeostasis may be associated with hypothyroidism during pregnancy; however, studies on this topic are lacking. In this study, we used metabolomics and proteomics to investigate the metabolic profile of hypothyroidism in the first half of pregnancy and its correlation with Th17/Treg balance.

## 2 Materials and methods

### 2.1 Research target

Pregnant women with regular peripartum coverage at the Obstetrics Outpatient Clinic of the Third Affiliated Hospital of Zhengzhou University from March 2024 to June 2024 were selected; 30 pregnant women with hypothyroid and 30 healthy pregnant women who met the inclusion criteria were recruited and included in the hypothyroidism group (H) and control group (C), respectively.

Inclusion criteria: 1) all pregnant women were ≤20 weeks of gestation; 2) the thyroid function of pregnant women in the hypothyroidism group was in accordance with the 2022 Guidelines for the Prevention and Management of Thyroid Diseases in Pregnancy and Childbirth and the reference range for hypothyroidism in pregnancy (TSH >4.0 mIU/L) of the Clinical Laboratory of the Third Affiliated Hospital of Zhengzhou University. Pregnant women in the control group had normal thyroid function and no other obstetric complications.

Exclusion criteria: 1) age <18 years or ≥35 years; 2) combined with other pregnancy complications; 3) artificial insemination, assisted reproduction technology/assisted conception; 4) twin and above pregnancies; 5) severe anxiety or depression; 6) currently taking antithyroid medication or thyroid medication substitution; 7) severe systemic diseases such as liver diseases or malignant tumors; 8) current infection or history of combined chronic inflammation and other autoimmune diseases; 9) pregnancy combined with hyperlipidemia or other comorbidities affecting the metabolic level of the body.

Ethical statement: This study was approved by the Medical Ethics Committee of the Third Affiliated Hospital of Zhengzhou University (Ethics No. 2024–284). All enrolled participants were aware of the risks and rights associated with the experiment, participated voluntarily, and signed informed consent forms.

### 2.2 Blood sample and cord blood collection and storage

Blood samples were collected from all pregnant women on the day of enrollment. All pregnant women fasted for 8–12 h before sample collection. Approximately 5 mL of blood was drawn from the elbow vein using a sterile syringe needle and placed in ethylenediaminetetraacetic acid (EDTA) and sodium heparin anticoagulant tubes (2.5ml each). Randomly selected 10 women from both group and collected their cord blood using EDTA tubes (3ml) during birth. Immediately after sampling, the blood samples were stored in a refrigerator at 4 °C. The blood samples in EDTA anticoagulated tubes were centrifuged (4 °C, 2000 rpm, 10 min) within 2 h and after centrifugation, the upper serum layer was aspirated using a sterile pasteurized pipette, placed in a freezing tube, and stored in a -80 °C refrigerator.Maternal blood collected in EDTA anticoagulant tubes was used for metabolomic and proteomic analyses, while umbilical cord blood was used for ELISA validation. Maternal blood collected in sodium heparin tubes was utilized for flow cytometry and cytokine experiments.

### 2.3 Data collection

Data on gestational week, age, body mass index (BMI), serum-free T4 (FT4), thyrotropin (TSH), antithyroid peroxidase antibodies (TPOAb), C-reactive protein (CRP), fasting blood glucose, and hemoglobin levels were collected from all pregnant women at enrollment. Upon enrollment, patients in the hypothyroidism group first provided blood samples and clinical data. In accordance with clinical guidelines and ethical guidelines, they then subsequently received oral levothyroxine therapy. Patients were monitored through follow-up, and clinical data on pregnancy-related comorbidities and delivery complications were collected. This included the incidence of conditions such as gestational diabetes mellitus, preterm labor, gestational hypertension, gestational anemia, and intrahepatic cholestasis during pregnancy.

### 2.4 Metabolomics analysis and data processing

For all participants in this study (30 pregnant women in the hypothyroidism group and 30 in the control group) maternal peripheral blood was collected in EDTA anticoagulant tubes at enrollment for metabolomic analysis. A Waters UPLC I-Class Plus (Waters, USA) in tandem with a Q Exactive high-resolution mass spectrometer (Thermo Fisher Scientific, USA) was used for metabolite separation and detection.

Chromatographic conditions: The chromatographic column used was a BEH C18 column (1.7 μm 2.1*100 mm, Waters, USA). The mobile phases in positive ionization mode were an aqueous solution containing 0.1% formic acid (liquid A) and methanol containing 0.1% formic acid (liquid B), and the mobile phases in negative ionization mode were aqueous solutions containing 10 mM ammonium formate (liquid A) and 95% methanol containing 10 mM ammonium formate (liquid B).

Mass spectrometry conditions: A Q Exactive mass spectrometer (Thermo Fisher Scientific, USA) was used for primary and secondary mass spectrometry data acquisition. The mass spectrometry was performed in the range of 70~1050, with a primary resolution of 70, 000, AGC of 3e6, and a maximum injection time (IT) of 100 ms. In accordance with the parent ion intensity, Top3 was selected for fragmentation, and the secondary information was collected with a secondary resolution of 17, 500, AGC of 1e5, a maximum IT of 50 ms, and fragmentation energy (IT, fragmentation energy) of 50 ms. The secondary resolution was 17, 500, the AGC was 1e5, the maximum IT was 50 ms, and the fragmentation energy (stepped once) was set to 20, 40, and 60 eV. The ion source parameters were set to 40 for the sheath gas flow rate, 10 for the auxiliary gas flow rate, and 10 for the spray voltage (spray voltage [|KV|]). |KV| was set to 3.80 for positive ion mode and 3.20 for negative ion mode. The capillary temp was set to 320 °C, and the aux gas heater temp was set to 350 °C. The parameters were set as follows: sheath gas flow rate at 40, aux gas flow rate at 10, |KV| at 3.80 for positive ion mode and 3.20 for negative ion mode, capillary temp at 320 °C, and aux gas heater temp at 350 °C.

Mass spectrometry data were imported into Compound Discoverer 3.3 (Thermo Fisher Scientific, USA) software and analyzed in conjunction with the BMDB (UW Metabolome Database, BGI Metabolome Database), mzCloud database, and ChemSpider online database. After analyzing the mass spectrometry data, a data matrix containing information on metabolite peak areas and identification results was obtained, and the table was further analyzed and processed.

### 2.5 Proteomics analysis and data processing

Eighteen patients from each group were randomly selected for proteomic analysis. The random selection procedure was conducted as follows: During the screening within the hypothyroidism group, a statistical analysis of the enrolled cohort was performed using Excel and assigned sequential identification numbers according to the order of enrollment. The RAND function was used to generate unique random numbers between 0 and 1, which were assigned to all participants in the hypothyroidism group. These random numbers were then sorted, leading to the selection of 18 corresponding random values. The INDEX function was subsequently applied in combination with the RAND function to display the identification numbers associated with the selected random values in Excel. The same procedure was used to randomly select 18 patients from the control group.Mass spectrometry data were collected for 36 samples using a TimsTOF Pro instrument in the data-independent acquisition (DIA) mode. The experimental procedure was as follows: protein extraction and enrichment were performed using a C18 column after extraction. After enrichment and quality control, an equal amount of peptide was extracted from each sample and mixed into one tube with 20 μg of peptide. A Shimadzu LC-20AD liquid chromatography system was used to take the mixed samples. Peptide separation was carried out using a 5 μm, 20 cm x 180 μm Gemini C18 column as a separator, and further separation was carried out using a 5 μm 4.6 x 250 mm Gemini C18 column as a separator. High-pH RP separation was performed to obtain DDA mode data. Downstream DDA data were analyzed using MaxQuant’s integrated Andromeda engine to create a spectral library. For large-scale DIA data, the mProphet algorithm was used to analyze the data for quality control. After completing the correction, MSstats derived from the bioconductor repository were used for difference analysis. Differential proteins were screened according to the criteria of multiplicity of differences >1.2 and Qvalue <0.05 as significant differences, and a series of functional analyses were performed on the differentially expressed proteins.

### 2.6 ELISA

Eighteen pregnant women with hypothyroidism and ≤20 weeks gestation and 18 normal pregnant women were selected as the validation cohort, and blood samples from the validation cohort were collected and stored using the same procedure. The validation cohort does not overlap with the main study cohort, and shares the same inclusion and exclusion criteria. From the main study cohort, ten pregnant women with hypothyroidism who delivered at our hospital were randomly selected to provide umbilical cord blood at delivery, using the same randomization method as applied in the proteomics screening. The same selection procedure was used for the control group participants.

The cord blood samples and validation cohort’s blood samples were thawed at room temperature and subsequently validated for selected metabolites and proteins using ELISA. The ELISA kits were from MEIMIAN Crop (Jiangsu, China). Human sphingosine ELISA kit (Catalog: MM-61057H1, sensitivity 120ng/L-4400ng/L) and Human BH3 interacting domain death agonist ELISA kit (Catalog: MM-0379H1, sensitivity 3pg/ml-150pg/ml) were used. The serum was diluted within the linear range of each assay following the manufacturer’s recommendations. The absorbance was measured at 450 nm using a Bioteck EPOCH enzyme-labeling instrument. Standard curves were drawn, and protein concentrations were calculated using ELISACALC software.

### 2.7 Flow cytometry

At enrollment, maternal peripheral blood was collected in sodium heparin tubes from all participants in both the hypothyroidism group (n=30) and the control group (n=30). Th17 was quantitatively measured using intracellular cytokine staining: after incubation at 37 °C 5% CO2 for 4 h the cell surface labeling antibody APC-CY7-CD3 (Clone:SP34-2) with FITC-CD4 (Clone:RPA-T4) was added. After incubation for 20 min in dark, erythrocyte lysate was added and incubated further for 25 min. Subsequently, centrifugation was performed at 500 g at 4 °C, and the supernatant was discarded. A fixed membrane-breaking solution was added, and the cells were incubated for 30 min in the dark. After washing, the APC-IL17 (Clone:SCPL1362) antibody was added for intracellular staining, and the cells were incubated for 30 min in the dark. Th17 cells were defined as CD3^+^CD4^+^IL-17^+^ lymphocytes. Treg cells were quantitatively measured using cell surface staining, and cell surface-labeling antibodies APC-CY7-CD3, FITC-CD4, APC-CD127 (Clone:HIL-7R-M21), and PE-CD25 (Clone:M-A251) were added to the peripheral blood and incubated in the dark. Erythrocyte lysates were incubated, centrifuged, washed, and resuspended in phosphate buffer. Treg cells were defined as CD3^+^CD4^+^CD25+CD127- lymphocytes. The samples were analyzed using flow cytometry. Data analysis was performed using the FlowJo software (Tree Star, Ashland, OR, USA). All antibodies were purchased from BD Biosciences (Franklin Lakes, NJ, USA).

### 2.8 Cytokine detection

The levels of IL-2, IL-6, IL-10, and TNF-a were measured using a Human Cytokine Kit (Jiangxi Sage Biotechnology Co., Ltd.) based on the flow fluorescence technique. The kit contains microspheres encapsulated with antibodies specific to IL-2, IL-6, IL-10, and tumor necrosis factor-alpha (TNF-α). The capture microsphere mixture was mixed with 25 μL of serum, and then the mixture was incubated with 25 μL of fluorescently labeled detection antibody for 2.5 h at 20–25 °C in the dark. The beads were washed and resuspended in phosphate buffer saline, and the samples were analyzed using flow cytometry.

Detection limits of the Kit is as followed: IL-2(0.2-5000pg/ml), IL-6(2.4-5000pg/ml), IL-10(1.22-5000pg/ml), TNF-α(0.5-5000pg/ml).

### 2.9 Statistical analysis

All statistical analyses of metabolomic and proteomic data were performed using metaX (21). Graphpad Prism 8.2 (GraphPad, La Jolla, CA, USA) was used for the statistical analysis of clinical indicators and flow cytometry. Measurement information that conformed to normal distribution was described as mean ± standard deviation, and a t-test was used for comparison between groups. Measures that did not meet the normal distribution were described as medians and quartiles, and the Mann–Whitney t-test was used for comparisons between groups. Categorical variables are described as frequencies, and differences between groups were determined using the chi-square test. Correlations were analyzed using Spearman’s analysis. Significance was set at P < 0.05.

## 3 Results

### 3.1 Clinical data of the hypothyroidism group and the control group

Overall, 30 pregnant women in the hypothyroidism group and 30 pregnant women in the control group were included in this study. As shown in **Table 1**, no significant differences were observed between the hypothyroidism and control groups in terms of age, BMI, gestational week, hemoglobin level, and fasting blood glucose level (P >0.05).

**Table 1:** Comparison of clinical data between hypothyroidism group and control group.

Clinical data	Hypothyroidism(n=30)	Control(n=30)	*P* value
Age (year)	30.27±3.373	29.53±3.421	0.407
BMI(kg/m^2^)	23.38±3.389	22.86±3.524	0.564
Gestation week(week)	13.80±4.817	13.03±3.899	0.501
Fasting blood glucose(mmol/L)	4.751±0.740	4.597±0.343	0.305
Hemoglobin(g/L)	117.7±11.46	121.8±9.806	0.139

### 3.2 Metabolomics

**Figure 1:**
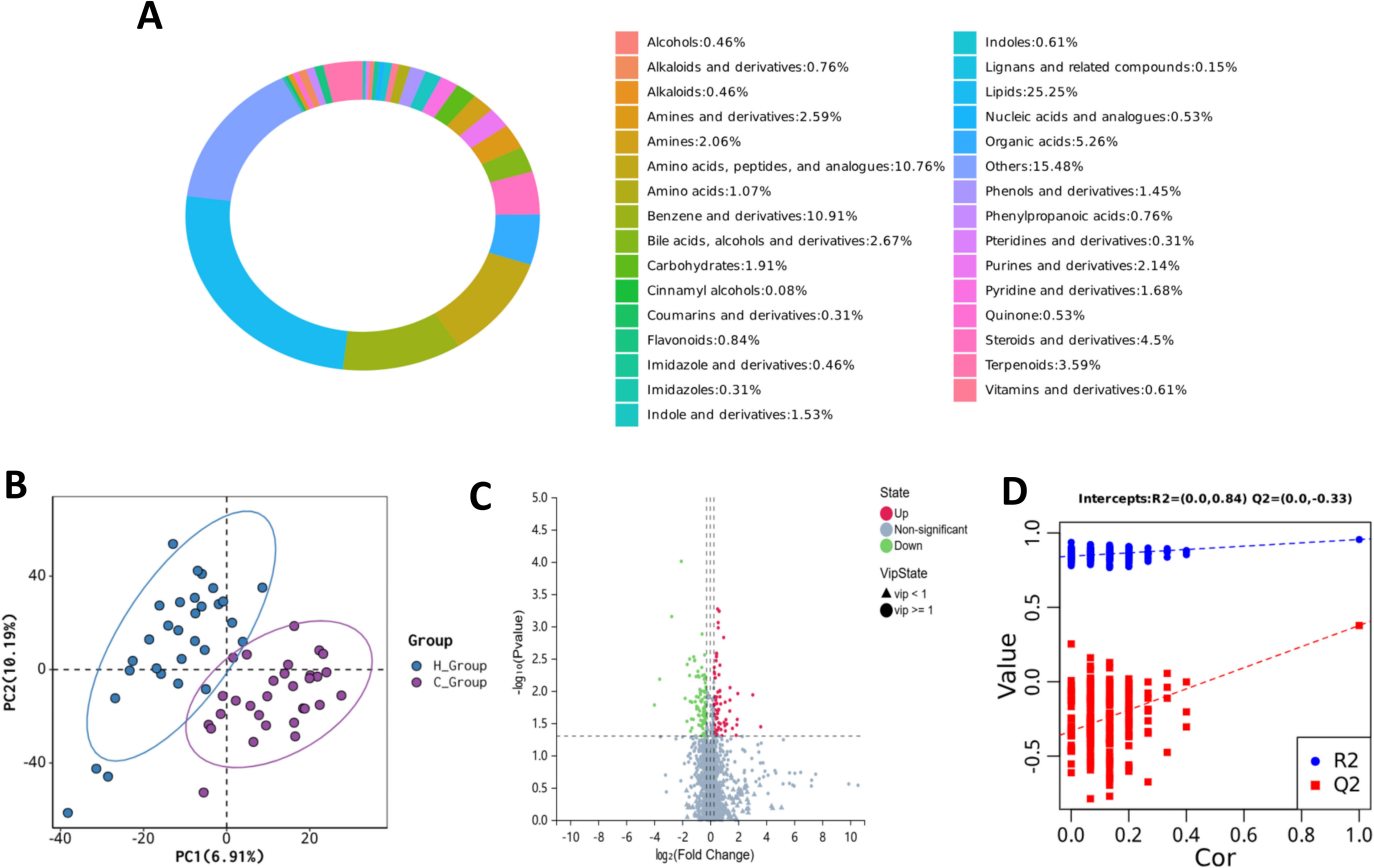
**(A)** Metabolite classification ring diagram: Different colors in the diagram indicate different metabolite classification entries, and the percentage indicates the percentage of the number of metabolites in that category to the number of all metabolites for which classification information is available; **(B)** Metabolomics partial least squares discriminant analysis (PLS-DA) diagram; **(C)** Metabolomics volcano diagram: The horizontal coordinate represents the log2-converted fold change (FC), and the vertical coordinate represents the -log10-converted q-value. Circles indicate metabolites with a VIP ≥1, triangles are metabolites with VIP <1. Downregulated significantly different metabolites are shown in green, upregulated significantly different metabolites in red, and non-significant metabolites in gray. **(D)** Permutation test: The two points in the upper right corner represent the R² and Q² values of the original model, while the point on the left shows the permutation test result.The metabolomics analysis included 30 samples per group (hypothyroidism vs. control), with all data processed using the MetaX software for statistical analysis.

The metabolite categorization loop (**Figure 1A**) showed that lipids accounted for 25.25% of all detected metabolites, making them the metabolite species with the largest percentage. Partial least squares discriminant analysis (PLS-DA) showed that the hypothyroidism group could be separated from the control group, indicating differences in metabolic profiles between the two groups (**Figure 1B**). Fold change (FC) ≥1.2, VIP ≥1, and P value <0.05 were used as parameters to screen the metabolites and plot the volcano diagrams, and the results shown in **Figure 1C** indicated a total of 183 differential metabolites between the two groups, with 75 metabolites significantly upregulated in the hypothyroidism group, 108 metabolites significantly downregulated, and sphingosine (Sph) was significantly upregulated.The permutation test results show no signs of overfitting (**Figure 1D**).

### 3.3 Proteomics

**Figure 2:**
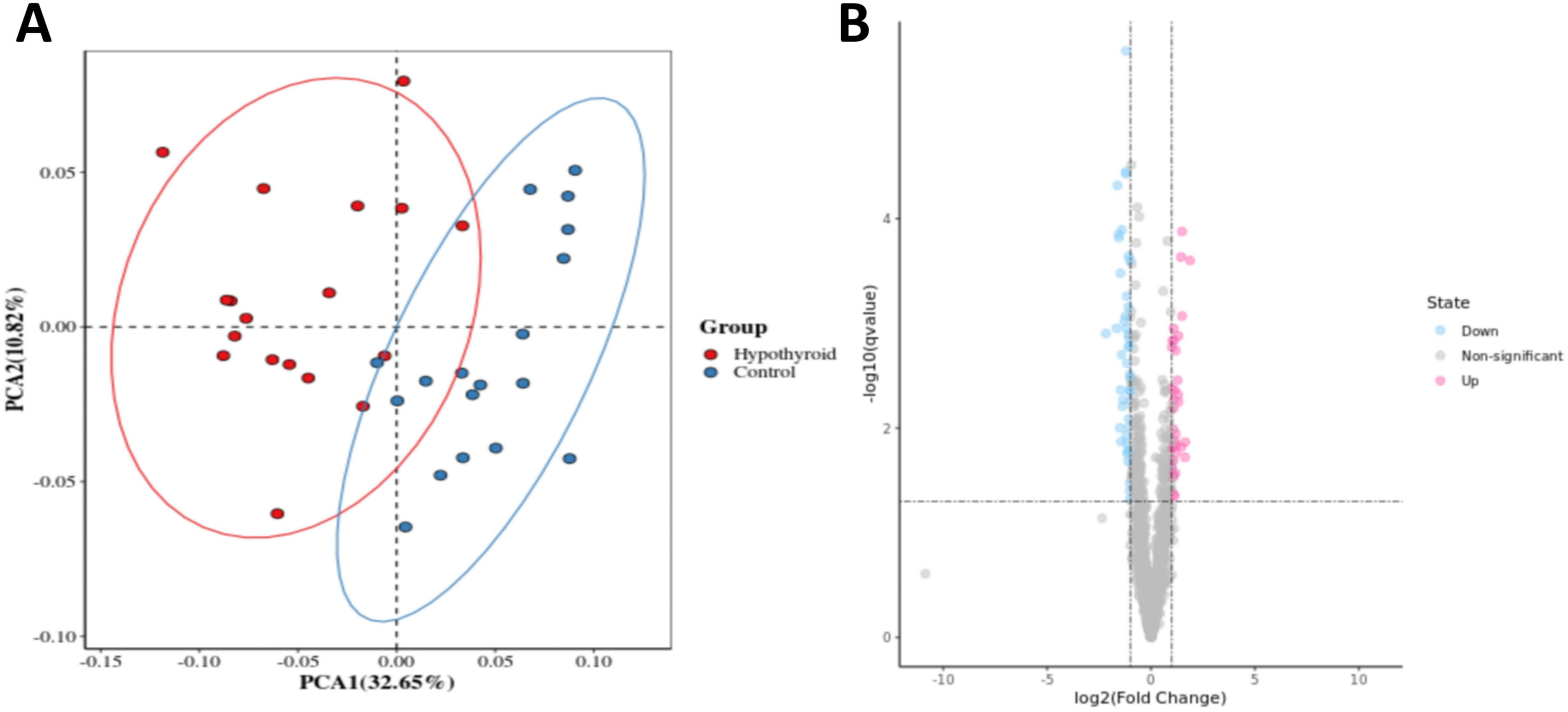
**(A)** Proteomics PLS-DA plot; **(B)** Proteomics volcano plot with downregulated significantly different proteins in blue, upregulated significantly different proteins in red, and non-significant proteins in gray.The proteomics analysis included 30 samples per group (hypothyroidism vs. control), with all data processed using the MetaX software for statistical analysis.

As shown in **Figure 2A**, the PLS-DA plot showed only a small overlap between the hypothyroidism and control groups, with most samples being separated, suggesting that the proteomic characteristics of the hypothyroidism group differed from those of the control group. FC ≥1.2 and P-value <0.05 were the conditions for the screening of differential proteins. Subsequently, a volcano plot of the differentially expressed proteins was drawn, and the results are shown in **Figure 2B**. Seventy-nine differentially expressed proteins were identified between the two groups, with 35 significantly upregulated, 44 significantly downregulated, and BH3 interacting domain death agonist (BID) significantly upregulated.

### 3.4 Multi-omics pathway association analysis

**Figure 3:**
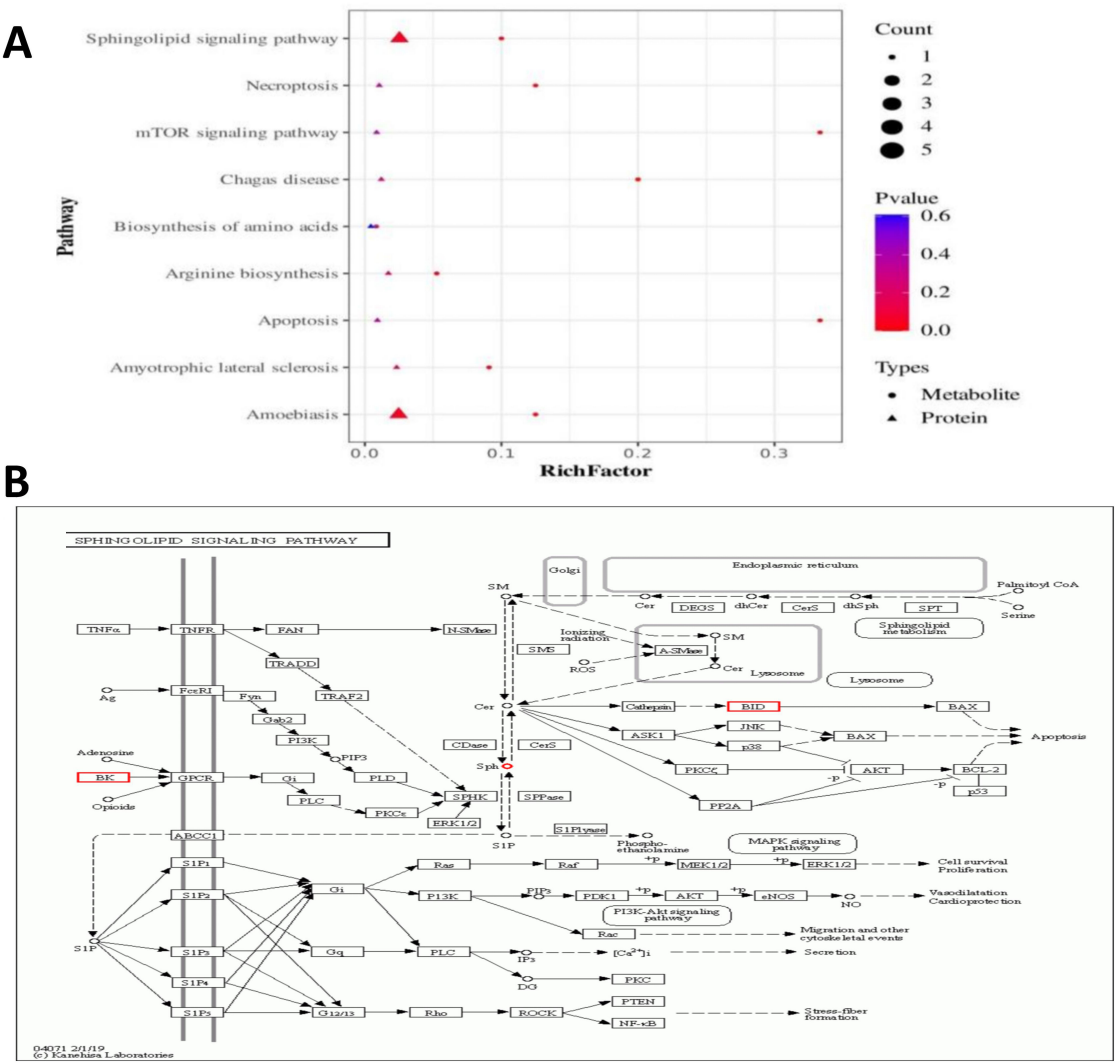
**(A)** Enrichment bubble graph of KEGG pathway for differential metabolites and differential proteins together. The x-axis is the enrichment ratio, and the y-axis is the KEGG pathway. Circles are differential metabolites, and triangles are differential proteins. The size of the graph indicates the number of metabolites or proteins annotated to a particular KEGG pathway, and the color represents the enrichment significance value (P-value), with a redder color representing a smaller P-value. **(B)** In the co-annotation graph of the sphingolipid signaling pathway, circles are the differential metabolites detected on the pathway, red color represents the upregulation of the differential metabolite, boxes are the differential proteins detected on the pathway, red color represents the upregulation of the differential protein. Sph and BID were highlighted in red.

Combined metabolomics and proteomics were performed for the Kyoto Encyclopedia of Genes and Genomes (KEGG) pathway analysis (**Figure 3A**), and the results showed that the sphingolipid signaling pathway (SSP) and ameobiasis pathway were significantly enriched in both histologies. Combined with the biological significance, the SSP was selected to draw a multi-omics pathway co-annotation map. As shown in **Figure 3B**, the upregulated differential metabolite Sph and the differential protein BID were co-enriched in the SSP, suggesting a regulatory relationship between Sph and BID. In addition, **Figure 3A** shows that the differential metabolites were significantly enriched in the necroptosis and apoptosis pathways.

### 3.5 ELISA

**Figure 4:**
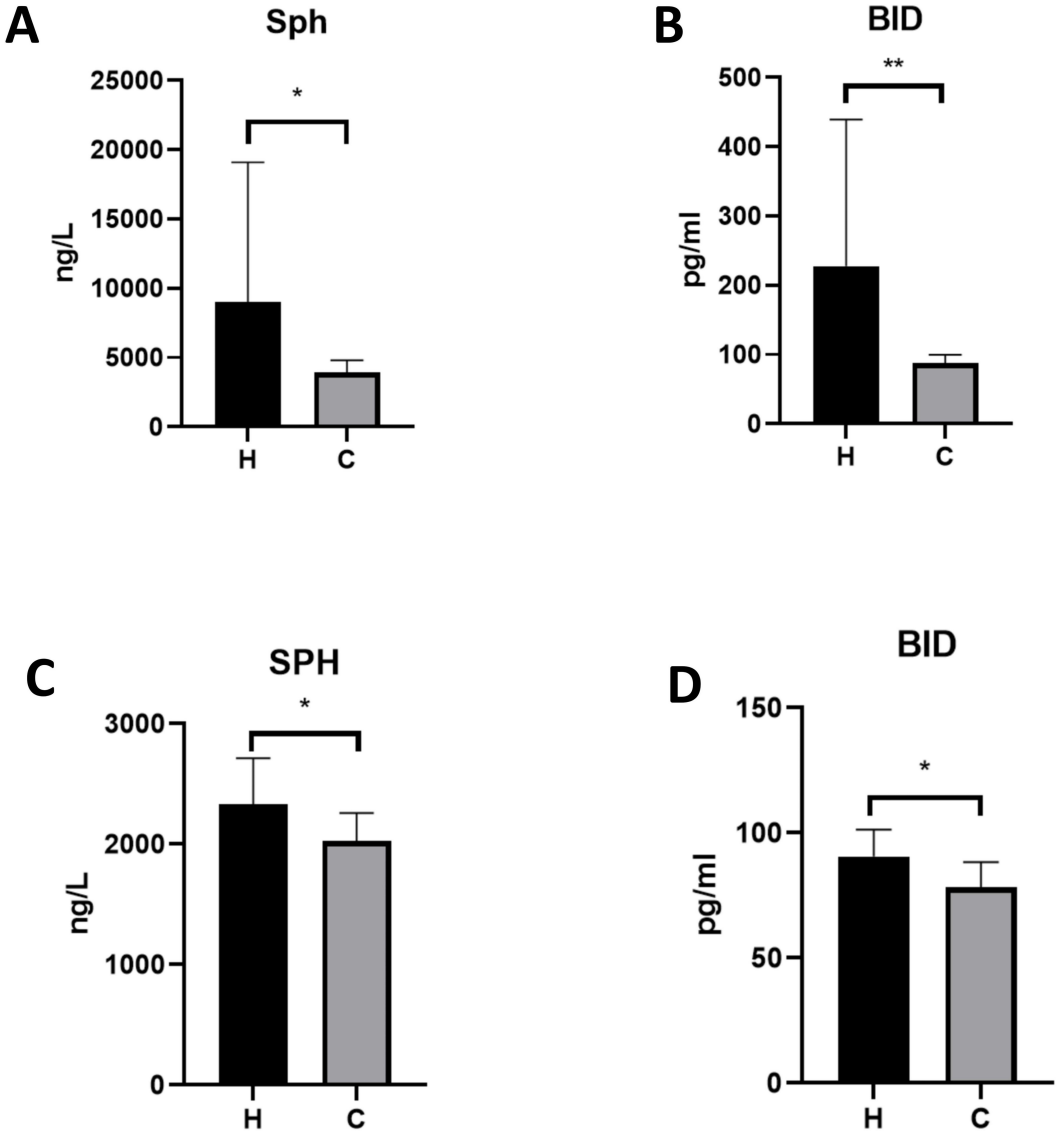
Bar plots of protein levels in different populations measured by ELISA. Serum levels of **(A)** Sph **(B)** BID and levels of Sph **(C)** BID **(D)** in cord blood were measured in the hypothyroidism group (H) and the control group (C).The ELISA analysis included 18 samples per group (hypothyroidism vs. control). Statistical comparisons were performed using t tests, with error bars in the figure representing standard deviation. * is p< 0.05 ** is p<0.01.

As shown in **Figure 4**, Sph and BID levels in the serum samples of the validation cohort and the cord blood samples were measured using ELISA, and both Sph and BID levels were significantly higher in the hypothyroidism group than in the control group.

### 3.6 Th17, Treg cells, and cytokine assay results

**Figure 5:**
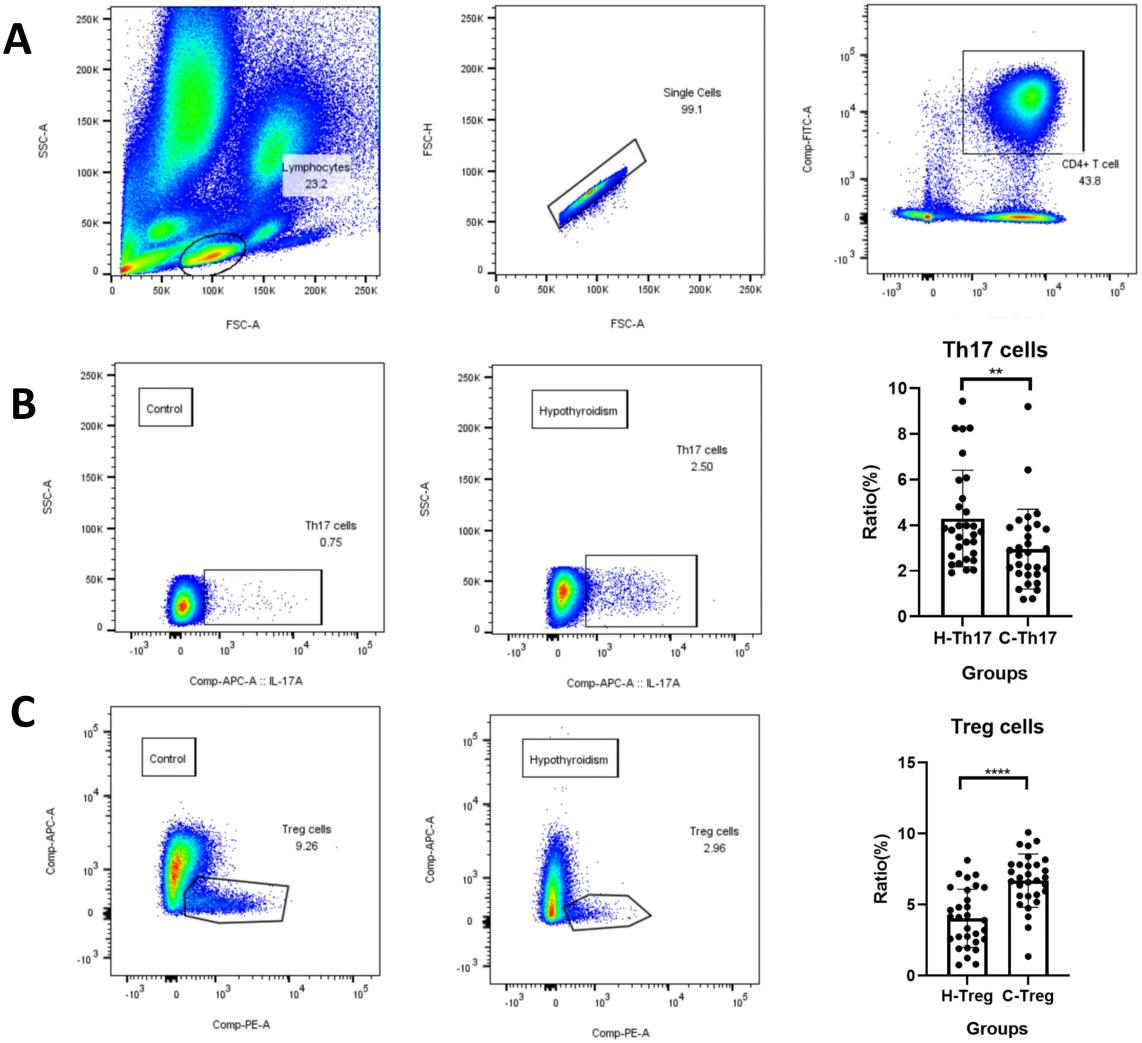
Detection of Th17, Treg cells using flow cytometry. **(A)** Flow cytometry gating strategy; **(B)** Th17 cell percentage; **(C)** Treg cell percentage.The flow cytometry analysis included 30 samples per group (hypothyroidism vs. control). Statistical comparisons were performed using t test and Mann-Whitney t test, with error bars in the figure representing standard deviation. ** is p< 0.01 **** is p< 0.0001.

Peripheral blood Th17 and Treg cells were detected in both groups of pregnant women using flow cytometry. The gating strategy is shown in **Figure 5A**. Lymphocytes co-expressing CD3 and CD4 were identified as CD3^+^CD4^+^ T cells. Th17 cells were defined as IL-17^+^ cells within the CD3^+^CD4^+^ T cell population, whereas Treg cells were identified as CD25^+^CD127^-^ cells within the same parent population. As shown in (**Figures 5B, C**) and **Table 2**, TSH, TPOAb, CRP, percentage of Th17 cells, Th17/Treg ratio, IL-2, IL-6, IL-10 and TNFα levels increased significantly in the hypothyroidism group compared to the control group, and FT4 levels and percentage of Treg cells decreased significantly.

**Table 2:** Comparison of Th17, Treg cell percentage and Th17/Treg ratio between two groups.

T cell/ cytokines	Hypothyroidism(n=30)	Control(n=30)	P value
TSH(mIU/L)	6.298±3.642	1.586±0.849	0.000^*^
FT4(mIU/L)	13.27±3.393	16.59±2.680	0.000^*^
TPOAb(U/ml)	65.80±99.02	12.80±5.342	0.005^*^
CRP(mg/L)	4.866±3.347	2.909±2.999	0.020^*^
Th17(%)	4.297±2.117	2.954±1.748	0.010*
Treg(%)	4.041±2.052	6.695±1.872	0.000*
Th17/Treg	0.976(0.591, 1.990)	0.384(0.251, 0.560)	0.000*
IL-2(pg/mL)	1.715(0.535, 3.148)	0.125(0.000, 1.070)	0.000*
IL-6(pg/mL)	7.228±6.298	3.930±5.869	0.040*
IL-10(pg/mL)	3.875(1.080, 6.433)	1.105(0.680, 2.303)	0.004*
TNF-α(pg/mL)	2.300(0.708, 6.443)	0.700(0.180, 1.305)	0.006*

* is p value<0.05.

### 3.7 Pregnancy comorbidities, complications and pregnancy outcomes in the hypothyroidism group and the control group

As shown in **Table 3**, the probability of developing gestational diabetes mellitus(GDM) in the hypothyroidism group was significantly higher than that in the control group, and the probabilities of developing gestational hypertension, preterm delivery, anemia, intrahepatic cholestasis of pregnancy and placental abruption during pregnancy were not significantly different from those of the control group. Neonatal weight in the hypothyroidism group was significantly lower than in the control group. There were no significant differences between the two groups in the incidence of intrauterine growth restriction or neonatal length.

**Table 3:** Number of patients with pregnancy comorbidities and complications in hypothyroidism group and control group.

Pregnancy Complications and outcomes	Hypothyroidism(n=30)	Control(n=30)	*P* value
gestational diabetes mellitus (n)	7	1	0.023*
gestational hypertension (n)	1	1	1.000
preterm delivery (n)	1	0	1.000
Anemia (n)	4	3	1.000
intrahepatic cholestasis of pregnancy (n)	0	0	1.000
Placentalabruption (n)	0	0	1.000
Intrauterine growth restriction (n)	1	0	1.000
neonatal weight (g)	3091±370.9	3329±387.4	0.018*
neonatal length (cm)	50.43±2.176	50.70±1.705	0.599

* is p value<0.05.

### 3.8 Correlation analysis

**Figure 6:**
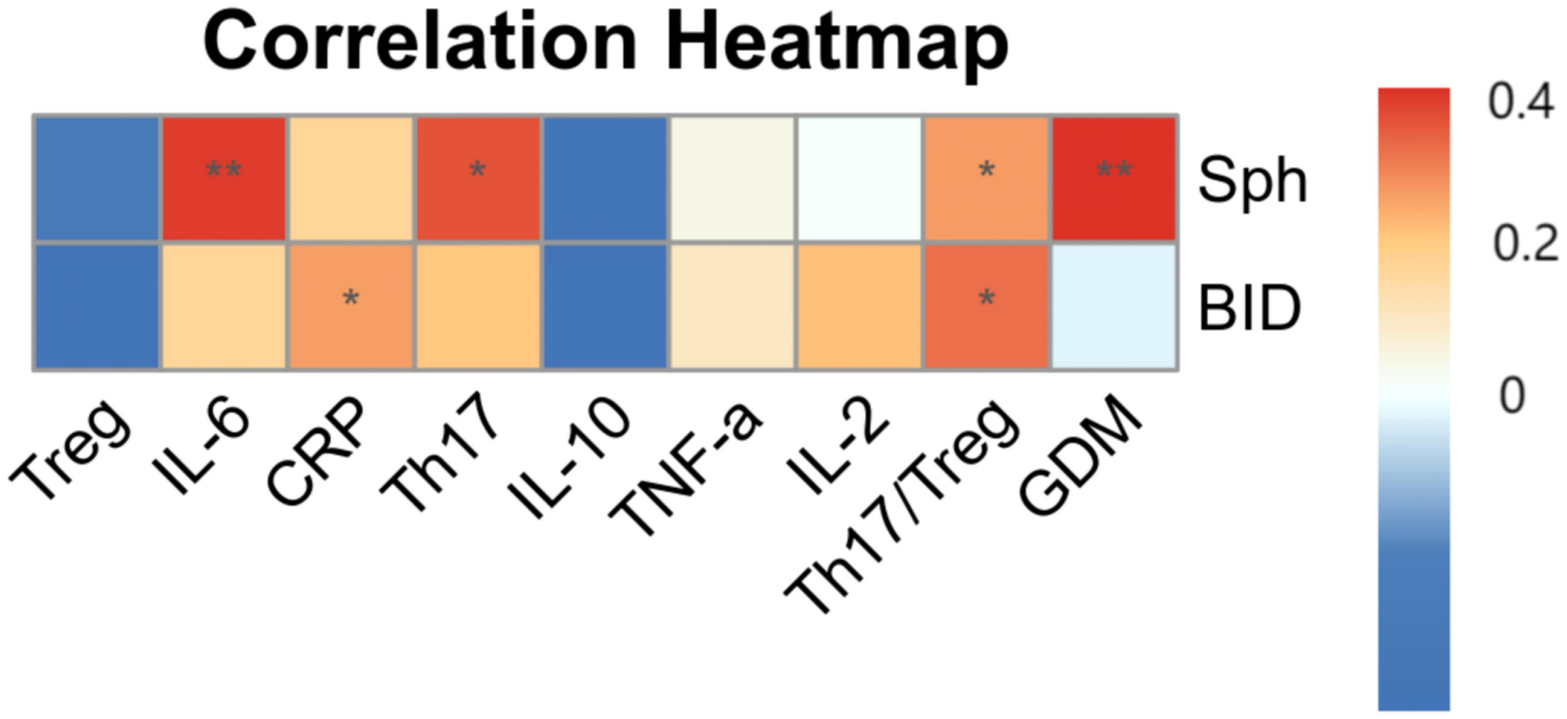
Correlation heatmap between bimarkers, flow cytometry, cytokines and pregnancy complication: Heatmap displaying correlations between biomarkers and flow cytometry data, cytokine levels, and pregnancy complications. Red indicates positive correlations, blue represents negative correlations, with color intensity corresponding to the absolute value of the correlation coefficient. Asterisks indicate statistical significance (*p < 0.05). ** is p<0.01.

Correlations between the potential biomarkers screened against the flow cytometry results, cytokine levels, and pregnancy complication were statistically analyzed using Spearman correlation analysis. As shown in **Figure 6**; **Table 4**, BID is positively correlated with CRP. Sph and BID positively correlated with Th17/Treg ratio, and Sph positively correlated with Th17, IL-6 levels and gestational diabetes.

**Table 4:** Correlation coefficients and p value of significantly correlated variables.

Variable 1	Variable 2	r value	P value
BID	CRP	0.340	0.042
Sph	Th17/Treg	0.343	0.041
BID	Th17/Treg	0.384	0.021
Sph	Th17	0.413	0.012
Sph	IL-6	0.433	0.008
Sph	GDM	0.445	0.007

## 4 Discussion

Hypothyroidism is a common endocrine disorder occurring during pregnancy. Until 20 weeks of gestation, the mother is the main provider of thyroid hormones for fetal growth and development, and hypothyroidism in the first half of pregnancy leads to an elevated risk of pregnancy complications and adverse pregnancy outcomes. Autoimmune disorders may be involved in the development of hypothyroidism during pregnancy; however, the exact mechanism is unknown. A combination of metabolomics and proteomics can be used to explore disease pathogenesis at the molecular level. In this study, we used metabolomics combined with proteomics to investigate the metabolic profile of hypothyroidism in the first half of pregnancy and its correlation with Th17/Treg balance.

No significant difference was observed between the general information of the hypothyroidism and control groups. Using metabolomics, we found 183 differential metabolites between the two groups, with lipids being the metabolite species with the largest proportion. Sph was significantly upregulated in the hypothyroidism group compared to the control group. Sph can be converted into ceramide via a remedial pathway catalyzed by ceramide synthase (CERS1-6), which can inhibit cellular proliferation and promote apoptosis (22, 23). The upregulation of Sph may disrupt sphingolipid metabolism and thus affect thyroid function (24), which is consistent with the findings of previous studies (13). However, a study by Babenko et al. found that altering the thyroid status of rats did not affect free Sph content (25), which is inconsistent with our results. The reasons for this discrepancy may be different study subjects, gestational statuses, and study methods. When Sph is upregulated, ceramides are increased, which may lead to an elevated intestinal inflammatory response and disruption of the intestinal immune environment (26), affecting the thyroid-gut axis (27), which is involved in developing hypothyroidism.

Quantitative analysis of protein differences revealed that the level of BH3 interacting BID was significantly higher in the hypothyroidism group than in the control group. BID plays a key role in the mitochondrial apoptotic pathway and enhances apoptotic signaling (28, 29). Vasconcelos et al. (30) found that thyroid hormones are associated with the upregulation of BID, which aligns with our results, and that the upregulation of BID promotes lipid exchange by binding to phospholipids to form soluble nanosized lipoprotein particles that bind and sequester anti-apoptotic Bcl-2 proteins. This process may lead to a decrease in cytoprotective activity and contribute to the apoptosis of thyroid cells, thereby inducing hypothyroidism during pregnancy.

Multi-omics analysis revealed that Sph and BID were co-enriched in the SSP by KEGG pathway analysis. We propose that the role played by the upregulation of Sph and BID in hypothyroidism during the first half of pregnancy may be as follows: the propensity for maternal oxidative stress increases during pregnancy (31) inducing the production of reactive oxygen species (ROS) (28). When the Sph level is upregulated, ROS can modulate the pathway of remedial conversion of intracytoplasmic ceramides, leading to an increase in ceramides (32, 33), which activates the release of histone B (34), hydrolyzing upregulated BID expression into an active truncated BID (tBID). tBID translocates to the mitochondrial outer membrane, leading to the enrichment of Bcl-2-associated X-protein in the mitochondrial outer membrane, followed by the promotion of mitochondrial outer membrane permeabilization, the formation of the mitochondrial outer membrane pore, and release of apoptotic proteins from the mitochondrial membrane (29, 35). This promotes apoptotic necrosis of thyroid cells in thyroid tissue, induces a proinflammatory environment, and enhances dysregulated immune responses involved in the development of hypothyroidism (36).

We further observed significantly elevated levels of both sphingosine and BID in the umbilical cord blood of the hypothyroidism group compared to the controls. This suggests that elevated maternal sphingosine and BID in gestational hypothyroidism may cross the placental barrier via the umbilical cord blood, inducing sphingolipid metabolic disturbances in both the placenta and the fetus (24). Disrupted fetal sphingolipid metabolism can upregulate apoptosis signaling pathways in placental cells, potentially leading to fetal growth restriction (37, 38). This may explain the significantly lower birth weight observed in newborns from the hypothyroidism group. However, no significant differences were found between the two groups in the incidence of intrauterine growth restriction or neonatal length. This discrepancy may be attributable to factors such as the limited sample size and the fact that women in the hypothyroidism group received thyroxine treatment after diagnosis and sample collection.

Flow cytometry analysis revealed an increased Th17 cell proportion, a decreased regulatory T cell (Treg) count, and an elevated Th17/Treg ratio in the hypothyroidism group. Autoimmune thyroid disease (AITD) represents the primary cause of hypothyroidism (15), with Hashimoto’s thyroiditis (HT) being its most common form, pathologically characterized by lymphocytic infiltration and follicular destruction (39). Compared to healthy controls, HT patients demonstrate significantly higher Th17 cell levels and serum IL-17A concentrations, along with substantially reduced Treg cell numbers (40). Previous studies have consistently shown that restoring the Th17/Treg balance can mitigate thyroid tissue damage and ameliorate autoimmune thyroid inflammation (41, 42). Therefore, we propose that Th17/Treg imbalance may represent one underlying mechanism contributing to thyroid dysfunction.

We found that CRP, IL-2, IL-6 and TNF-α levels were increased in pregnant women with hypothyroidism in the first half of pregnancy. CRP levels increased in the hypothyroidism group, indicating the occurrence of a systemic inflammatory response and an elevated release of proinflammatory factors in pregnant women with hypothyroidism during pregnancy (43). An increase in CPR may suggest the presence of upregulated oxidative stress in pregnant women with hypothyroidism (44). The upregulation of IL-6 may lead to an increase in the number of cells activated by Th17 (28), shift the Th17/Treg balance in the direction of Th17, and increase the secretion of Th17-associated cytokine IL-17. This initiates the production of further proinflammatory cytokines, such as TNF-α, that may be involved in hypothyroidism in pregnancy through stimulation of the nuclear factor-κB signaling pathway that extensively damages thyroid cells, affects apoptosis, and alters the immune status (45). We observed that elevated IL-10 levels were accompanied by a reduction in Treg cell numbers. This phenomenon may be explained by following factors: The imbalance in the Th17/Treg ratio may disrupt the immune microenvironment, potentially activating myeloid-derived suppressor cells (MDSCs), which produce substantial IL-10 as a compensatory response to immune dysregulation (46–48). This chronic stimulation may induce functional exhaustion in Treg cells (49), leading to loss of immunosuppressive capacity and acquisition of pathogenic T cell features, such as those of Th1 cells, thereby aggravating autoimmune pathology (50). Furthermore, when lipid metabolism is disturbed, the secretion of IL-10 increases, altering the mitochondrial activity of Treg cells. The nutrients obtained by the cells are insufficient to meet their metabolic needs, inhibiting the proliferation and development of Treg cells (51); therefore, the ability of the body to resist autoimmunity is reduced (52).

Statistics on pregnancy comorbidities and complications between the two groups found that more pregnant women in the hypothyroidism group developed gestational diabetes than in the control group. Kent et al. found that a significant increase in the odds of GDM was observed when using a TSH threshold of >4.0 mIU/l (53), which is in accordance with our results. In people with hypothyroidism during pregnancy, impaired translocation of glucose transporter protein-4 may occur in the muscle and adipose tissues, leading to tissue resistance to insulin, promoting a state of insulin resistance in the body, and participating in the development of gestational diabetes mellitus (54). In addition, increased levels of anti-TPO antibodies may affect the development of gestational diabetes. Elevated TPOAb can spread through the placental barrier during pregnancy, affecting placental development and the inflammatory immune status of the placenta (55), which may lead to impaired placental β-HCG secretion and promote the development of insulin resistance, favoring the development of gestational diabetes mellitus (54).

Spearman’s correlation analysis revealed a significant positive correlation between Sph, Th17 cell count, Th17/Treg ratio, and IL-6 levels.BID is positively correlated with Th17/Treg ratio. This may be due to the fact that (1)The upregulation of Sph may lead to increased levels of its phosphorylated product, sphingosine-1-phosphate (S1P) (56). S1P can activate signal transduction in CD4^+^ T cells through the sphingosine signaling pathway, which may enhance IL-17 expression. This process promotes Th17 cell differentiation, thereby disrupting the Th17/Treg balance and increasing the secretion of related inflammatory cytokines. As a result, thyroid cells become more susceptible to BID-mediated apoptosis, ultimately accelerating thyroid cell death and contributing to the pathogenesis of gestational hypothyroidism (57). (2) elevated Sph may lead to increased β-glucosylceramide production by upregulating ceramide production (58). The upregulation of BID expression may promote apoptosis (29), which increases the release of β-glucose ceramide from dying cells (59). β-glucosylceramide acts as a mincle ligand and promotes Th17 cell proliferation in a mincle-dependent manner (60). This leads to a shift in the Th17/Treg balance, which promotes inflammatory and autoimmune responses and is involved in the development of hypothyroidism in pregnancy (45). (3) Sph is upregulated, and S1P may be increased (56). S1P binds to Th17-expressed sphingosine-1-phosphate receptor 1 in the thymus and mediates the transfer of more Th17 cells from the thymus to the peripheral circulation, increasing the circulating Th17/Treg balance (61). The increased number of Th17 cells, activated by rising IL-6 levels, secrete cytokines that influence the autoimmune status and are involved in the development of hypothyroidism in pregnancy (62, 63). Moreover, Sph is positively associated with gestational diabetes mellitus. Tan et al. showed that sph levels are elevated in populations with gestational diabetes mellitus, which is consistent with our findings (64). The upregulation of Sph, which may lead to an increase in ceramide, may be involved in the development of gestational diabetes mellitus by promoting apoptosis and injury of pancreatic β-cells. Additionally, it may impair insulin-stimulated glucose uptake and glycogen synthesis in skeletal muscle and adipocytes *in vivo* by blocking the translocation of glucose transporter protein-4, resulting in insulin resistance, affecting the development of gestational diabetes (64, 65).

A significant positive correlation was observed between BID and CRP levels. Elevated CRP levels may indicate the presence of oxidative stress in pregnant women (44). BID can promote a pro-oxidative environment in mitochondria, and its upregulation may induce or exacerbate maternal oxidative stress (66). This could subsequently promote apoptosis, amplify dysregulated immune responses, and contribute to the development of gestational hypothyroidism.

This study had some limitations. The results of this study may be biased owing to the limited sample size. Therefore, we plan to further investigate the pathogenesis of hypothyroidism in pregnancy through approaches such as *in vitro* functional studies, animal models, and large-scale cohort analyses. In accordance with standard clinical and ethical guidelines, patients in the hypothyroidism group received levothyroxine tablets after enrollment. This treatment may have influenced the umbilical cord blood findings and pregnancy outcomes, and further experimental studies will be needed to confirm these results. Furthermore, This study represents preliminary research; the effects of Sph and BID on fetal outcomes and their long-term implications for both mother and child require further investigation in future studies.

In summary, this study explored the metabolic features of hypothyroidism during the first half of pregnancy using metabolomics and proteomics. The findings indicate that the upregulation of sphingomyelin may influence BID through the SSP to promote BID-mediated apoptosis, which is involved in the onset and development of hypothyroidism during pregnancy. Additionally, we examined the correlation between the upregulation of sph and BID and the Th17/Treg balance from an immune perspective to explore the possible involvement of autoimmunity in hypothyroidism during the first half of pregnancy, providing a new theoretical basis for understanding the mechanism of hypothyroidism in the first half of pregnancy.

## Data Availability

The datasets presented in this study can be found in online repositories. The names of the repository/repositories and accession number(s) can be found below: http://www.proteomexchange.org/, PXD058969 https://osf.io/kfmcn/?view_only=a076baadf44f4714aaf37869995f30ea, 10.17605/OSF.IO/KFMCN.
